# Regorafenib is effective against neuroblastoma in vitro and in vivo and inhibits the RAS/MAPK, PI3K/Akt/mTOR and Fos/Jun pathways

**DOI:** 10.1038/s41416-020-0905-8

**Published:** 2020-05-27

**Authors:** Divya Subramonian, Nikki Phanhthilath, Hannah Rinehardt, Sean Flynn, Yuchen Huo, Jing Zhang, Karen Messer, Qianxing Mo, Shixia Huang, Jacqueline Lesperance, Peter E. Zage

**Affiliations:** 1 Department of Pediatrics, Division of Hematology-Oncology, University of California San Diego, La Jolla, CA USA; 2 Department of Family Medicine and Public Health, University of California San Diego, La Jolla, CA USA; 3 Department of Medicine, Dan L. Duncan Cancer Center, Baylor College of Medicine, Houston, TX USA; 4 Peckham Center for Cancer and Blood Disorders, Rady Children’s Hospital, San Diego, CA USA; 5 Present Address: Department of Biostatistics & Bioinformatics, H. Lee Moffitt Cancer Center & Research Institute, 12902 Magnolia Drive, Tampa, FL 33612 USA

**Keywords:** Paediatric cancer, Targeted therapies

## Abstract

**Background:**

Regorafenib is an inhibitor of multiple kinases with aberrant expression and activity in neuroblastoma tumours that have potential roles in neuroblastoma pathogenesis.

**Methods:**

We evaluated neuroblastoma cells treated with regorafenib for cell viability and confluence, and analysed treated cells for apoptosis and cell cycle progression. We evaluated the efficacy of regorafenib in vivo using an orthotopic xenograft model. We evaluated regorafenib-mediated inhibition of kinase targets and performed reverse-phase protein array (RPPA) analysis of neuroblastoma cells treated with regorafenib. Lastly, we evaluated the efficacy and effects of the combination of regorafenib and 13-*cis*-retinoic acid on intracellular signalling.

**Results:**

Regorafenib treatment resulted in reduced neuroblastoma cell viability and confluence, with both induction of apoptosis and of cell cycle arrest. Regorafenib treatment inhibits known receptor tyrosine kinase targets RET and PDGFRβ and intracellular signalling through the RAS/MAPK, PI3K/Akt/mTOR and Fos/Jun pathways. Regorafenib is effective against neuroblastoma tumours in vivo, and the combination of regorafenib and 13-*cis*-retinoic acid demonstrates enhanced efficacy compared with regorafenib alone.

**Conclusions:**

The effects of regorafenib on multiple intracellular signalling pathways and the potential additional efficacy when combined with 13-*cis*-retinoic acid represent opportunities to develop treatment regimens incorporating regorafenib for children with neuroblastoma.

## Background

Neuroblastoma is the most common extracranial solid tumour of childhood, and children with high-risk neuroblastoma currently have long-term survival rates under 40% despite intensive, multimodal treatment regimens that include chemotherapy, surgical tumour resection, autologous stem cell transplantation, radiation therapy, and maintenance immunotherapy combined with 13-*cis*-retinoic acid.^1,2^ Children with high-risk neuroblastoma also frequently suffer from treatment-resistant tumours and disease relapse, and children with recurrent or refractory neuroblastoma respond poorly to additional chemotherapy.^3–5^ New treatments and therapeutic combinations directed at relevant targets are needed for these children to reduce relapse rates and improve survival.

Neuroblastoma cells and primary tumours express a wide range of growth factors and receptor kinases that represent potential therapeutic targets.^6–20^ However, therapeutic agents directed against individual targets or individual signalling pathways have had limited success in children with neuroblastoma, suggesting that therapies that target multiple relevant targets concurrently are likely to demonstrate increased efficacy.

Regorafenib (BAY 73-4506) is an orally active, diphenylurea multikinase inhibitor that has been FDA approved for the treatment of metastatic colorectal cancer, advanced gastrointestinal stromal tumours (GIST) and progressive hepatocellular carcinoma after prior sorafenib therapy.^21–24^ Regorafenib potently inhibits the RET, RAF1, VEGFR2, c-kit, VEGFR1, PDGFRβ, VEGFR3 and FGFR1 kinases, with IC_50_ values of 1.5 nM, 2.5 nM, 4.2 nM, 7 nM, 13 nM, 22 nM, 46 nM and 202 nM, respectively, in cell-free assays.^21^ Regorafenib furthermore has demonstrated significant antitumour activity in a range of preclinical models, including models of paediatric solid tumours.^25,26^ We therefore hypothesised that regorafenib would demonstrate efficacy against neuroblastoma preclinical models via the combined inhibition of multiple critical surface receptors and intracellular signalling pathways.

## Methods

### Cells and culture conditions

The neuroblastoma cell lines used in this study have been previously utilised by our laboratory^27–29^ and were purchased from American Type Culture Collection (ATCC, www.atcc.org) or were generously provided by Shahab Asgharzadeh (Children’s Hospital Los Angeles, Los Angeles, CA), Susan Cohn (The University of Chicago Children’s Hospital, Chicago, IL), Jill Lahti (St. Jude Children’s Research Hospital, Memphis, TN), John Maris (Children’s Hospital of Philadelphia, Philadelphia, PA) or the Children’s Oncology Group (COG) Cell Culture and Xenograft Repository (www.cogcell.org). Cell lines were grown at 37 °C in 5% CO_2_ in appropriate media (Invitrogen, Carlsbad, CA) supplemented with 10% heat-inactivated foetal bovine serum (FBS) (Life Technologies, Grand Island, NY), L-glutamine, sodium pyruvate and non-essential amino acids (Sigma-Aldrich, St. Louis, MO). All cell lines were authenticated by DNA profiling prior to use. Cell line features are listed in Supplemental Table 1.

### Therapeutic agents

Regorafenib (BAY 73-4506) was generously provided by Bayer, AG (Berlin, Germany). A 10 mM stock solution was generated in 100% DMSO (Sigma-Aldrich) and stored at –20 °C. Regorafenib was diluted in phosphate-buffered saline (PBS) immediately before use. For in vivo studies, regorafenib was diluted to a final concentration of 5 mg/mL in 1,2-propanediol, polyethylene glycol 400 and pluronic F68 (42.5/42.5/15) (Sigma-Aldrich) as previously described.^21,30^ All compound preparations were stored at room temperature in the dark and used the same day. 13-*cis*-retinoic acid (Sigma-Aldrich) was diluted directly into media prior to use.

### Cell viability assays

Human neuroblastoma cell lines were tested for sensitivity to regorafenib in vitro using a modified methyl tetrazolium (MTT, Sigma-Aldrich) assay.^28,29^ 0.5–1.0 × 10^4^ exponentially growing cells in 135-μL media were plated in individual wells in 96-well plates, and 24 h later, regorafenib or vehicle alone was added to each well at specified concentrations using an automated drug delivery system (Biomek Automated Laboratory Workstation, Beckman Coulter, Inc., Fuller, CA). After 72 h of continuous drug exposure, 15 µL of 5 mg/ml MTT was added to each well, and the plates were incubated for 4 h at 37 °C. Media was replaced with 150 µL of DMSO, and the optical density (OD) was measured at 550 nm using a microplate spectrophotometer (Anthos Labtec Instruments, Wals, Austria). Relative cell viability was calculated by subtracting the background OD of media alone and then dividing by the OD of control wells. Replicates of three wells were used for each drug concentration, and assays were repeated on separate days. Concentration that inhibits 50% (IC_50_) values were derived using best-fit trendlines, and values calculated using the relevant curve-fit equations as previously published.^27–29^

For regorafenib and 13-*cis*-retinoic acid combination studies, cells were plated as above and treated with regorafenib alone, 5 μM 13-*cis*-retinoic acid alone or combinations of 5 μM 13-*cis*-retinoic acid and increasing concentrations of regorafenib for 72 h. Cell viability was determined as above. Analysis of variance (ANOVA) was used to investigate potential synergy by including an interaction term between 13-*cis*-retinoic acid (CRA) and regorafenib across the tested neuroblastoma cell lines.

### Cell confluence assays

Continuous live-cell imaging was performed as previously described.^31^ Briefly, neuroblastoma cells were plated in 96-well plates at seeding densities between 10,000 and 25,000 cells/well and treated with regorafenib alone and in combination with 13-*cis*-retinoic acid as above. The plates were placed into the IncuCyte^®^ Zoom^TM^ continuous live-cell imaging system (Essen Bioscience, Ann Arbor, MI), and phase-contrast images were taken every 6 h at 10× magnification for 72 h. Cell confluence was calculated using IncuCyte^®^ analysis software. Replicates of at least three wells were used for each experimental condition, and the assay was performed at least three independent times. Cell growth curves were generated from the calculated percent cell confluence. IC_50_ values were derived using best-fit trendlines, and the values were calculated using the appropriate curve-fit equations as above.

### Caspase 3/7 apoptosis assays

Neuroblastoma cells were plated at 2000 cells/well in 96-well plates and placed into the IncuCyte^®^ Zoom^TM^ as above. After 24 h, cells were treated with 2.5 µM, 5 µM or 10 µM regorafenib, and 5 μM IncuCyte^®^ Caspase 3/7 Green Reagent (Essen Bioscience) was added. Phase-contrast and fluorescence images were taken every 6 h, with four non-overlapping images taken per well at 10× magnification for 72 h as above. Average green object counts (each representing individual cells with increased caspase activity) per field were generated and normalised to control. Replicates of at least three wells were used for each experimental condition.

### Western blots

Neuroblastoma cells were plated in 6-well plates or 10-cm tissue culture dishes in media with 10% FBS at ~80% confluency and allowed to adhere overnight. Cells were then treated with either regorafenib or vehicle for designated times. Treated cells were harvested, washed with cold PBS and lysed using RIPA buffer supplemented with Pierce Protease inhibitor and phosphatase inhibitor (Life Technologies).

Protein concentration was measured using a BCA Protein Assay Kit (Thermo Fisher Scientific, San Diego, CA). Equal amounts of protein were loaded onto 4–12% Bis-Tris gels (Invitrogen, Carlsbad, CA) with MOPS SDS running buffer (Life Technologies) and transferred to PVDF membranes (Thermo Fisher) using the iBlot2 Dry Blotting transfer system (Invitrogen) or using the Invitrogen Mini Blot Module and Novex Transfer Buffer (Life Technologies).

Membranes were blocked in 5% BSA in TBST (TBS + 0.1% Tween-20) for 1 h at room temperature and then incubated overnight with primary antibodies (all from Cell Signaling, Danvers, MA, except as noted below) to total MEK (9126; 1:1000), phosphorylated MEK (9154; 1:1000), total ERK (4695; 1:1000), phosphorylated ERK (4370; 1:1000), total PDGFR-β (3175; 1:1000), phosphorylated PDGFR-β (4549; 1:500), total RET (3220; 1:1000), phosphorylated RET (3221; 1:500), total FGFR1 (9740; 1:1000), phosphorylated FGFR1 (2544; 1:500), GAPDH (5174; 1:2000), p38 MAPK (8690; 1:1000), total Akt (9272; 1:1000), phosphorylated Akt (4060; 1:2000), total S6 (2217; 1:1000), phosphorylated S6 (4858; 1:2000), c-Jun (9165; 1:1000), poly-ADP ribose polymerase (PARP) (9542; 1:1000), GATA3 (558656; 1:1000, BD Pharmingen, San Diego, CA), Vinculin (ab1290002; 1:1000, Abcam, Cambridge, MA) or β-actin (AS316; 1:20000, Sigma-Aldrich).

All antibodies were diluted in blocking buffer to achieve the specified concentrations. Membranes were then washed three times with PBS-T (PBS + 0.1% Tween-20) and incubated for 1 h at room temperature with HRP-conjugated anti-rabbit (W401B; 1:5000, Promega, Madison, WI) or anti-mouse (W402B; 1:5000, Promega) IgG secondary antibodies. The signal was visualised using Amersham ECL Prime Luminol Enhancer and Peroxide Solution (GE Healthcare, Piscataway, NJ), and membranes were developed using SuperSignal™ West Pico Plus Chemiluminescent Substrate (Thermo Fisher). Membranes were exposed to film using Amersham Biosciences Hypercassettes and Denville Scientific HyBlot CL Films (Thomas Scientific, Swedesboro, NJ), and film was developed in an ECOMAX™ X-ray film processor (Protec, Oberstenfeld, Germany).

### Flow cytometry

Neuroblastoma cells were plated as above and treated with regorafenib or vehicle for 24 h. Cells were then harvested and washed with ice-cold PBS and centrifuged for 5 min at 500 *g*. The cell pellet was resuspended in 200 μL of PBS/0.1% FBS, and 4 mL of ice-cold 70% ethanol was added to the cells dropwise. The fixed cells were then incubated overnight at –20 °C. Cells were resuspended and rehydrated in PBS, treated with 100 μg/ml RNAse and then stained with 50 μg/ml propidium iodide. The cell pellet was then incubated in 1 mL of propidium iodide (PI) solution (50 μg/mL PI (Sigma-Aldrich), 100 μg/mL RNaseA and 0.1% Triton X-100 in PBS) at 37 °C for 1 h. Flow cytometry was performed on a FACSCanto-II flow cytometer (BD Biosciences, Franklin Lakes, NJ), and the results were analysed using FlowJo flow cytometry analysis software (v10, Tree Star Inc., Ashland, Ore) to determine the distribution of different cell cycle phases.

### Reverse phase protein arrays

Reverse-phase protein array (RPPA) assays were carried out as described previously with minor modifications.^32,33^ SK-N-SH, SK-N-AS, IMR-32 and SK-N-BE(2) neuroblastoma cells were plated as above and treated with 5 μM regorafenib, 5 μM 13-*cis*-retinoic acid, the combination of 5 μM regorafenib plus 5 μM 13-*cis*-retinoic acid or vehicle alone for 24 h. Cells were harvested, and protein lysates were prepared from treated and untreated cells using tissue protein extraction reagent (Pierce, Rockford, IL) supplemented with 450 mM NaCl and a mixture of protease and phosphatase inhibitors (Roche Life Science, San Francisco, CA). Protein levels were quantified as above, and protein lysates at 0.5 μg/μL were denatured in SDS sample buffer (Life Technologies) with 2.5% (vol/vol) 2-mercaptoethanol at 100 °C for 8 min. The Aushon 2470 Arrayer (Aushon BioSystems, Billerica, MA) with a 40-pin (185 μm) configuration was used to spot lysates onto nitrocellulose-coated slides (Grace Bio-Labs, Bend, OR) using an array format of 960 lysates/slide (2880 spots per slide) with each sample spotted as technical triplicates, including test and control lysates. The slides were processed as described,^32,33^ and probed with a set of 214 validated antibodies (https://www.bcm.edu/centers/cancer-center/research/shared-resources/antibody-based-proteomics) against total and phosphorylated proteins using an automated slide stainer Autolink 48 (Dako, Carpinteria, CA). Each slide was incubated with one specific primary antibody, and the negative control slide was incubated with only antibody diluent containing no primary antibody. Primary antibody binding was detected using a biotinylated secondary antibody followed by streptavidin-conjugated IRDye680 fluorophore (LI-COR Biosciences, Lincoln, NE). Total protein content of each spotted lysate was assessed by fluorescent staining with Sypro Ruby Protein Blot Stain according to the manufacturer’s instructions (Molecular Probes, Eugene, OR). Fluorescence-labelled slides were scanned on a GenePix AL4200 scanner, and the images were analysed with GenePix Pro7.0 (Molecular Devices, Sunnyvale, CA). Total fluorescence signal intensities of each spot were obtained after subtraction of the local background signal for each slide and were then normalised for variation in total protein, background and nonspecific labelling using a group-based normalisation method as described.^32,33^ Each image, along with its normalised data, was carefully evaluated for quality through manual inspection and control samples. Antibody slides that failed the quality inspection were either repeated at the end of the staining runs or removed before data reporting. Differentially expressed proteins across samples were determined by one-way ANOVA, and the expression values were log2-transformed and mean-centred for visualisation in heat maps using Java TreeView.

### Statistical analyses

The results from the RPPA analyses (above) were log transformed in base 2, and the average values of technical replicates (3 for each sample) were used for analysis. Analysis of variance (ANOVA) was used to compare treatment groups across the four tested neuroblastoma cell lines (see equation 1). *P* values for the main treatment effects across all cell lines were calculated for each protein. Proteins were tagged as differentially expressed between two groups if *p* < 0.05 for the main treatment effect. The Benjamini–Hochberg correction was used to correct raw p values, with a 25% false discovery rate (FDR).

1 $$\begin{array}{l}{\mathrm{Fit}} = {\mathrm{lm}}\left({\mathrm{protein}}\, \sim\, {\mathrm{cell}}{\_}{\mathrm{SKNAS}} + {\mathrm{cell}}{\_}{\mathrm{SKNSH}} + {\mathrm{cell}}{\_}{\mathrm{SKNBE}}\right.\\ \;\left.+ {\mathrm{cell}}{\_}{\mathrm{IMR32}} + {\mathrm{treat}}{\_}{\mathrm{main}} -1, {\mathrm{data}} = {\mathrm{data}}\right)\qquad\end{array}$$

To evaluate whether *MYCN* amplification has an effect on observed changes in protein expression, an interaction was added to the formula (see equation 2).

2 $$\begin{array}{l}{\mathrm{Fit}} ={\mathrm{lm}}\left({\mathrm{protein}} \sim{\mathrm{cell}}{\_}{\mathrm{SKNAS}} + {\mathrm{cell}}{\_}{\mathrm{SKNSH}} +{\mathrm{cell}}{\_}{\mathrm{SKNBE}} \right.\qquad\qquad\qquad\qquad\\ \left. +\,{\mathrm{cell}}{\_}{\mathrm{IMR32}} +{\mathrm{treat}}{\_}{\mathrm{main}} +{\mathbf{treat}}{\_}{\mathbf{main}}^*{\mathbf{AMP}} -1, {\mathrm{data}} ={\mathrm{data}}\right)\;\;\end{array}$$

Similarly, to evaluate whether known sensitivity to retinoic acid has an effect on the observed changes in protein expression, an interaction term was added to the formula (see equation 3).

3 $$\begin{array}{l}{\mathrm{Fit}} ={\mathrm{lm}}\left({\mathrm{protein}} \sim{\mathrm{cell}}{\_}{\mathrm{SKNAS}} + {\mathrm{cell}}{\_}{\mathrm{SKNSH}} +{\mathrm{cell}}{\_}{\mathrm{SKNBE}} \right.\qquad\qquad\quad\\ \qquad\;\,\left. +\, {\mathrm{cell}}{\_}{\mathrm{IMR32}} +{\mathrm{treat}}{\_}{\mathrm{main}} +{\mathbf{treat}}{\_}{\mathbf{main}}^*{\mathbf{SENS}} -1, {\mathrm{data}} ={\mathrm{data}}\right)\end{array}$$

Similar analyses were performed to compare the RPPA results obtained from cells treated with regorafenib to those treated with vehicle alone, with 13-*cis*-retinoic acid to vehicle alone, and to cells treated with regorafenib plus 13-*cis*-retinoic acid to vehicle alone. Additional similar analyses were performed using analysis of variance (ANOVA) to investigate potential synergy by including an interaction term between 13-*cis*-retinoic acid (CRA) and regorafenib across the tested neuroblastoma cell lines (as above).

### Animal experiments

To evaluate the efficacy of regorafenib against neuroblastoma tumours in vivo, 4- to 6-week-old female athymic nu/nu mice weighing 20–25 g were purchased from the UCSD Animal Care Program and housed in the UCSD Moores Cancer Center vivarium. Mice were anaesthetised with ketamine/medetomidine in vivarium procedure rooms, the left flanks were shaved and prepared in sterile fashion using betadine followed by 70% ethanol, and transverse incisions were performed to expose the left kidneys and adrenal glands. In total, 1 × 10^6^ SK-N-SH neuroblastoma cells engineered to constitutively express firefly luciferase were suspended in 5 μL of PBS and injected into exposed adrenal glands using a 27-gauge needle, which results in >90% of mice developing tumours.^28,29,34^ The peritoneum and the skin were then closed in two separate layers, and wound clips were placed. Animal body temperature was maintained during the surgery using Delta Phase Pads (Braintree Scientific), and buprenorphine was administered prior to the procedure as an analgesic, followed by additional doses as needed every 8–12 h after the procedure. After recovery from surgery, mice were placed in cages in their appropriate cage location. Wound clips were removed after 10 days. Tumour development and growth were monitored twice per week in mice anaesthetised by nasal isoflurane using the Xenogen Lumina system (Caliper Life Sciences, Hopkinton, MA) 10 min after intraperitoneal injections of 150 mg/kg D-luciferin (Caliper Life Sciences), and tumour volume was estimated using signal intensity (in p/s/cm^2^/sr). Three weeks after surgery at the estimated time of onset of tumour growth,^28,29,34^ mice were randomly separated into two groups. Mice in group 1 were gavage-fed once daily with vehicle alone, while the other group of mice was fed once daily with regorafenib at 30 mg/kg. Both groups of mice were treated for 14 days and then euthanised by CO_2_ followed by cervical dislocation. Tumours were harvested, weighed and photographed. Student’s t tests on log values of tumour volumes as measured by Lumina signal intensity and on final tumour weights were used to calculate p values for tumour growth. All mice were housed and treated according to protocols approved by the Institutional Animal Care and Use Committee at UCSD.

## Results

### Regorafenib reduces neuroblastoma cell viability and confluence and xenograft tumour growth

**Fig. 1:**
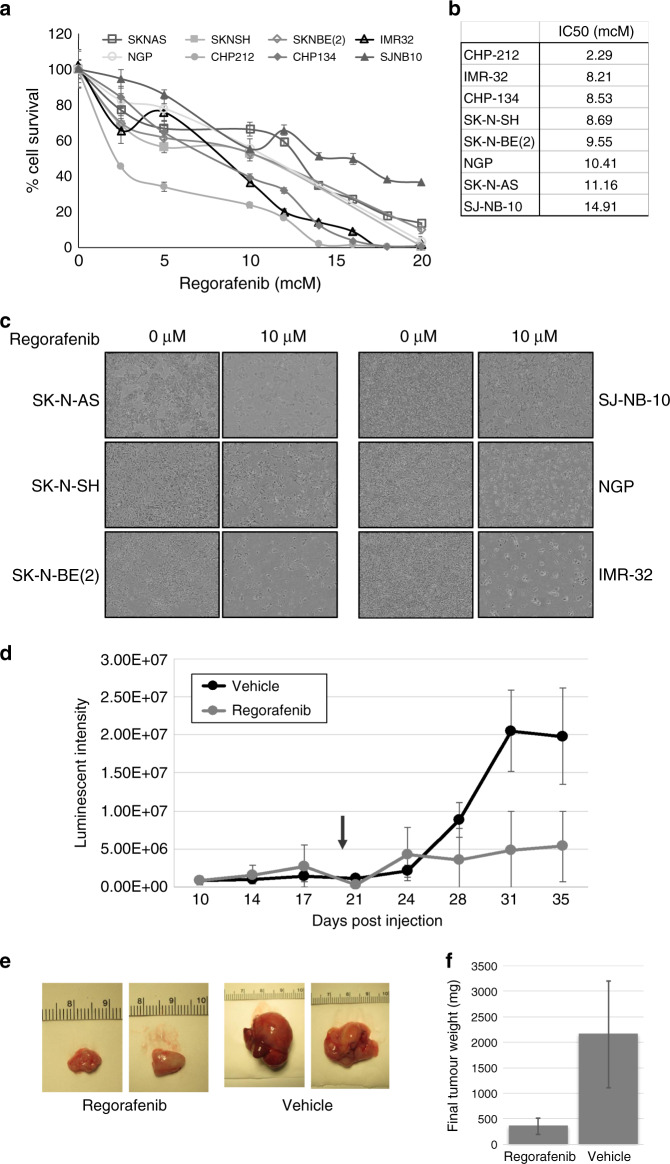
Regorafenib reduces neuroblastoma cell viability and xenograft tumour growth. **a** Neuroblastoma cell lines (SK-N-AS, SK-N-SH, SK-N-BE(2), IMR-32, NGP, CHP-212, CHP-134 and SJ-NB-10) were treated with increasing concentrations of regorafenib for 72 h, and cell viability was measured using MTT assays. Mean average values from triplicate experiments were then plotted against regorafenib dose levels. **b** IC_50_ values were calculated using curve-fit equations for each tested neuroblastoma cell line. **c** Images of untreated and treated neuroblastoma cells were taken at regular intervals, and representative images of untreated and treated neuroblastoma cells after 72 h are shown. **d** Mice with orthotopic adrenal SK-N-SH neuroblastoma xenograft tumours were treated with vehicle (*n* = 4) or regorafenib (*n* = 5). Tumour development and growth were monitored twice per week, and tumour volume was estimated using luminescent signal intensity (in p/s/cm^2^/sr). The arrow represents the start of regorafenib treatment, and changes in signal intensity were plotted over time (*p* = 0.10). **e** Tumours were harvested after 14 days of treatment, and representative final images for harvested tumours from untreated control and regorafenib-treated mice are shown. **f** Tumour weights from harvested tumours were obtained, and the average values were calculated, with significance determined by Student’s *t* test (*p* < 0.05).

To determine the efficacy of regorafenib against neuroblastoma cells, a panel of established human neuroblastoma cell lines representing a range of biological phenotypes (Supplemental Table 1) was tested for sensitivity in vitro to regorafenib using MTT assays and continuous live- cell imaging. IC_50_ values from the cell viability assays were calculated and ranged from 2.3 μM to 14.9 μM (Fig. 1a, b), suggesting that neuroblastoma cell lines demonstrate similar sensitivity to regorafenib. Regorafenib treatment also resulted in reduced neuroblastoma cell confluence as determined by continuous live-cell imaging, with IC_50_ values similar to those calculated from cell viability assays (Fig. 1c, Supplemental Fig. 1a, b). There were no significant differences in responses after 24, 48 or 72 h of exposure to regorafenib (Supplemental Fig. 2), and no apparent associations were observed between response to the drug and biologic or genetic features of individual cell lines.

To evaluate the efficacy of regorafenib against neuroblastoma tumours in vivo, neuroblastoma cells were injected into the adrenal glands of immunocompromised mice, and mice that subsequently developed tumours were treated with either vehicle alone or regorafenib. Regorafenib treatment was well tolerated with no weight loss or other apparent symptoms, and regorafenib-treated mice demonstrated reduced xenograft tumour growth rates compared to vehicle-treated controls (Fig. 1d). Final tumour size and weight were also significantly reduced in tumours from mice treated with regorafenib compared with vehicle-treated control tumours (Fig. 1e, f), with vehicle-treated tumours reaching an average final tumour weight of 2.16 g, and tumours from mice treated with regorafenib limited to an average final tumour weight of 0.35 g (*p* < 0.05).

### Regorafenib induces neuroblastoma cell apoptosis and cell cycle arrest

**Fig. 2:**
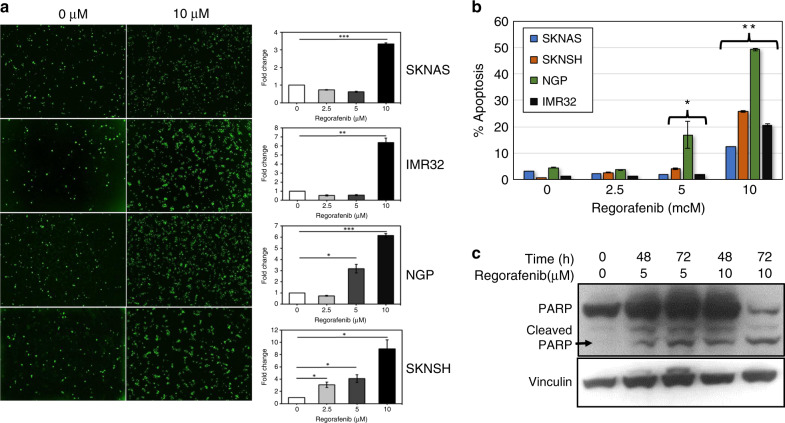
Regorafenib induces neuroblastoma cell apoptosis. SK-N-AS, IMR-32, NGP and SK-N-SH neuroblastoma cells were treated with 2.5, 5 or 10 µM regorafenib for 72 h, and were evaluated using a caspase activity assay. Images were obtained and were analysed using the IncuCyte Zoom^TM^, with individual green dots representing cells with increased caspase activity. **a** Images of untreated cells and of cells treated with 10 μM regorafenib for 72 h are shown. Average green dot counts per field were normalised to control cells, and fold change was determined by comparing average green object counts in treated cells with average counts in untreated cells. The final results were compared using Student’s t tests (*p < 0.05, **p < 0.01, ***p < 0.001). **b** The average percentage of apoptotic cells (as measured by green object count) compared with the total cell number determined by phase-contract imaging. Changes in the numbers of individual cells treated with regorafenib with increased caspase activity were normalised to controls and compared with untreated cells. The final results were compared using Student’s *t* tests (*p < 0.05, **p < 0.00001). **c** SK-N-SH neuroblastoma cells were treated with 5 µM or 10 µM regorafenib for 48 or 72 h, and treated cells were lysed and analysed by western blot for total and cleaved PARP.

To determine the mechanisms underlying the observed reduction in cell viability, cell confluence and tumour growth induced by regorafenib treatment, we performed caspase activity assays in neuroblastoma cells treated with regorafenib and in vehicle-treated control cells. Regorafenib treatment resulted in increased caspase activity in all tested cell lines in a dose-dependent fashion (Fig. 2a, b). We further evaluated cells treated with regorafenib for cleavage of poly-ADP ribose polymerase (PARP) by western blot, and found that PARP cleavage also increased in neuroblastoma cells after regorafenib treatment (Fig. 2c), further suggesting that regorafenib treatment leads to the induction of neuroblastoma cell apoptosis.

To determine whether regorafenib also induced changes in cell cycle progression, neuroblastoma cells were treated with increasing concentrations of regorafenib and analysed by flow cytometry for DNA content. Regorafenib treatment for 24 h resulted in an increase in the percentage of cells in the G0/G1 phase from 65.1% to 84.7% in SK-N-SH cells and from 44.5% to 62.9% in IMR-32 cells (Supplemental Fig. 3), demonstrating that regorafenib is effective against neuroblastoma cells via a combination of both induction of apoptosis and of cell cycle arrest.

### Regorafenib inhibits target kinases and intracellular signalling pathways

**Fig. 3:**
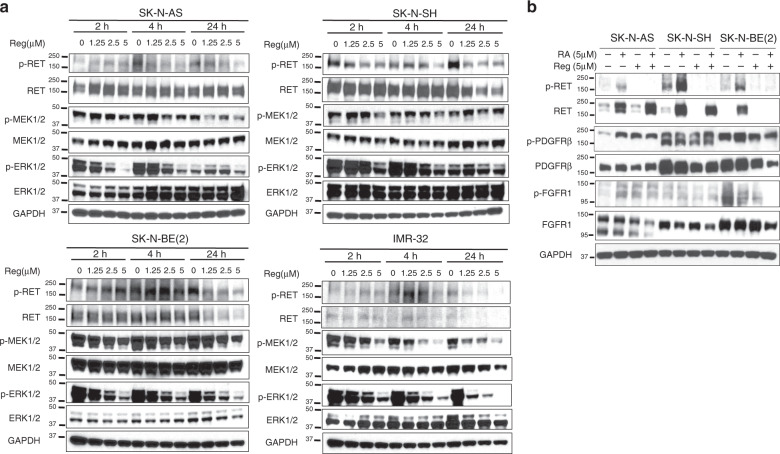
Regorafenib inhibits targets in neuroblastoma cells. **a** Neuroblastoma cells were treated with vehicle alone or increasing doses of regorafenib (Reg) for 2, 4 or 24 h, and the protein lysates were analysed by western blot for total and phosphorylated RET (RET, p-RET), MEK1/2 (MEK1/2, p-MEK1/2) and ERK1/2 (ERK1/2, p-ERK1/2). GAPDH was used as a loading control. **b** Neuroblastoma cells were treated with vehicle alone, 5 μM 13-*cis*-retinoic acid (RA), 5 μM regorafenib (Reg) or the combination of 13-*cis*-retinoic acid and regorafenib for 24 h, and the protein lysates were analysed by western blot for total and phosphorylated RET (RET, p-RET), total and phosphorylated FGFR1 (FGFR1, p-FGFR1) and total and phosphorylated PDGFRβ (PDGFRβ, p-PDGFRβ). GAPDH was used as a loading control.

Regorafenib has been demonstrated to inhibit multiple kinases, including the RET, VEGFR1–3, c-Kit, TIE-2, PDGFRβ, FGFR1, RAF1, BRAF and p38 MAPK kinases.^21^ To determine whether the efficacy of regorafenib was a result of inhibition of cell surface receptor kinases, we evaluated neuroblastoma cells after regorafenib treatment for inhibition of known receptor tyrosine kinase targets. Regorafenib treatment of neuroblastoma cells resulted in reduced phosphorylation of RET within 4 h of regorafenib exposure in a dose-dependent manner (Fig. 3a), with reduced total RET levels also observed in SK-N-SH and SK-N-BE(2) cells after 24 h of exposure (Fig. 3a, b). Regorafenib treatment also inhibited 13-*cis*-retinoic acid-induced RET phosphorylation, and led to reduced expression of FGFR1 and PDGFRβ, with incomplete inhibition of FGFR1 and PDGFRβ phosphorylation also noted in tested neuroblastoma cell lines (Fig. 3b, Supplemental Fig. 4).

To evaluate the effects of regorafenib on intracellular signalling downstream of RAF, we also evaluated neuroblastoma cells after regorafenib treatment for RAS-MAPK pathway signalling activity. Regorafenib treatment of neuroblastoma cells resulted in inhibition of signalling through the RAS-MAPK pathway, with reduced MEK and ERK phosphorylation in dose- and time-dependent fashion and minimal effects on total MEK and ERK levels after 24 h of exposure in most tested cell lines (Fig. 3a). However, no differences in the inhibition of known regorafenib targets were noted in tested cell lines that were more or less sensitive to regorafenib, suggesting that alternative pathways may mediate specific neuroblastoma cell sensitivity to regorafenib.

### Regorafenib induces changes in intracellular signalling pathway activity in neuroblastoma cells

**Fig. 4:**
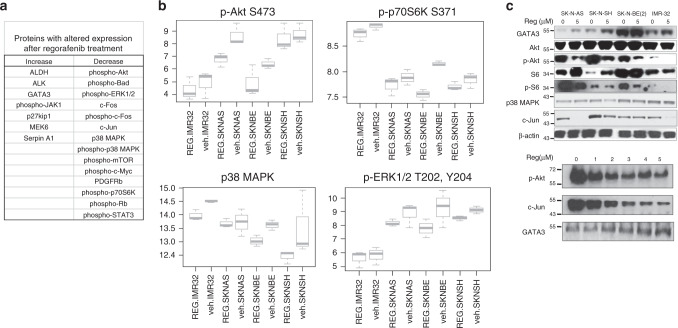
Regorafenib treatment alters protein expression and phosphorylation in neuroblastoma cells. Neuroblastoma cells were treated with either vehicle or 5 µM regorafenib for 24 h. Lysates were collected and analysed by reverse-phase protein array (RPPA). **a** The results were analysed, and proteins whose levels or phosphorylation were significantly increased or decreased (*p* < 0.05 for each using ANOVA) after drug treatment when compared with vehicle alone are shown. **b** Relative protein levels of phospho-Akt (p-AKT S473), phospho-p70S6K (p-p70S6K S371), p38 MAPK and phospho-ERK1/2 (p-ERK T202, Y204) were calculated as described, and are shown in regorafenib- treated (REG) and vehicle-treated (veh) neuroblastoma cells, with the results displayed separately for independent cell lines. **c** Neuroblastoma cells were treated with 5 μM regorafenib for 24 h, and cell lysates were analysed by western blot for GATA3, total Akt, phospho-Akt (p-Akt S473), total S6, phospho-S6 (p-S6 S235/236), p38 MAPK, c-Jun and β-actin levels (top). Neuroblastoma cells were treated with increasing concentrations of regorafenib for 24 h, and the protein lysates were analysed by western blot for p-Akt, c-Jun and GATA3 (bottom).

To identify additional signalling proteins and pathways altered by regorafenib treatment, we evaluated neuroblastoma cell lines with reverse-phase protein arrays (RPPA). RPPA analysis of lysates of untreated neuroblastoma cells and of cells treated with 5 μM regorafenib for 24 h identified 22 proteins with significant changes in expression and/or phosphorylation (*p* < 0.05 for each). Phosphorylation of Akt, mTOR, ERK, c-Fos, p38 MAPK and S6, among others, was decreased upon exposure to regorafenib (Fig. 4a, b), suggesting inhibition of multiple critical intracellular signalling pathways in a dose-dependent manner. Moreover, protein expression of ALK, MEK6 and GATA3 was increased after regorafenib treatment, suggesting potential mechanisms for neuroblastoma cell resistance to regorafenib. Protein expression changes observed by RPPA were validated independently by western blot analyses (Fig. 4c). Within these 22 identified proteins, 5 were found to have significant interaction between *MYCN* amplification and response to regorafenib, with levels of phosphorylated c-Fos, phosphorylated Bad and total levels of c-Jun having more significant decreases after regorafenib treatment in non-*MYCN*-amplified cells compared with those with *MYCN* amplification (Supplemental Fig. 5).

### Regorafenib combined with 13-*cis*-retinoic acid is effective against neuroblastoma cells and induces changes in intracellular signalling pathway expression and activity

**Fig. 5:**
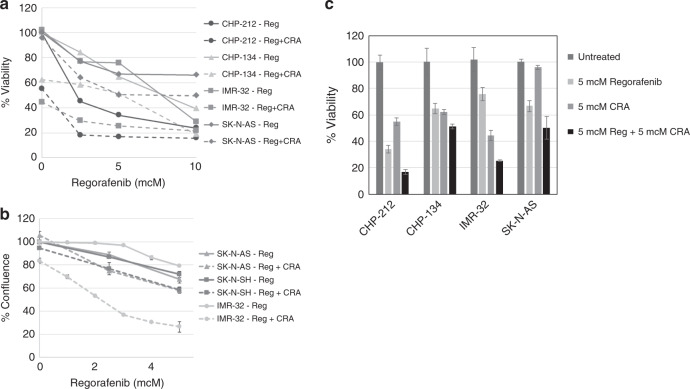
Efficacy of regorafenib combined with 13-*cis*-retinoic acid in neuroblastoma cells. Neuroblastoma cells were treated with increasing concentrations of regorafenib (Reg) alone or in combination with 5 µM 13-*cis*-retinoic acid (CRA) for 72 h. **a** Viability was determined by MTT assay, and mean average values from triplicate experiments were plotted against regorafenib dose levels. **b** Neuroblastoma cells treated with regorafenib and CRA were monitored by continuous live- cell imaging, and the percentage of normalised cell confluence was calculated for each cell line. Average values from triplicate experiments were plotted against regorafenib dose levels. **c** CHP-212, CHP-134, IMR-32 and SK-N-AS neuroblastoma cells were treated with 5 μM regorafenib, 5 μM CRA or the combination of regorafenib with CRA for 72 h, and cell viability was determined by MTT assay. Mean average values from triplicate experiments were plotted. The final results were compared using ANOVA (**a**, **c**). Potential synergy was investigated by including an interaction term between 13-*cis*-retinoic acid and regorafenib in ANOVA models, and the interaction term showed a non-significant additive effect in each case (*p* = 0.07).

We have previously shown that the combination of RET inhibition with 13-*cis*-retinoic acid, a vitamin A analogue currently used for maintenance therapy in children with neuroblastoma,^35^ demonstrated synergistic efficacy against neuroblastoma in preclinical models.^29,36^ To determine whether 13-*cis*-retinoic acid also enhanced the efficacy of regorafenib, neuroblastoma cell lines were treated with regorafenib alone and in combination with 13-*cis*-retinoic acid. In tested cell lines, the combination of regorafenib with 13-*cis*-retinoic acid was more effective than regorafenib alone over a range of regorafenib concentrations (Fig. 5a–c), and the combination was more effective in both retinoic acid-sensitive (SK-N-SH, SK-N-BE(2)) and retinoic acid-resistant (IMR-32, SK-N-AS) cell lines.

**Fig. 6:**
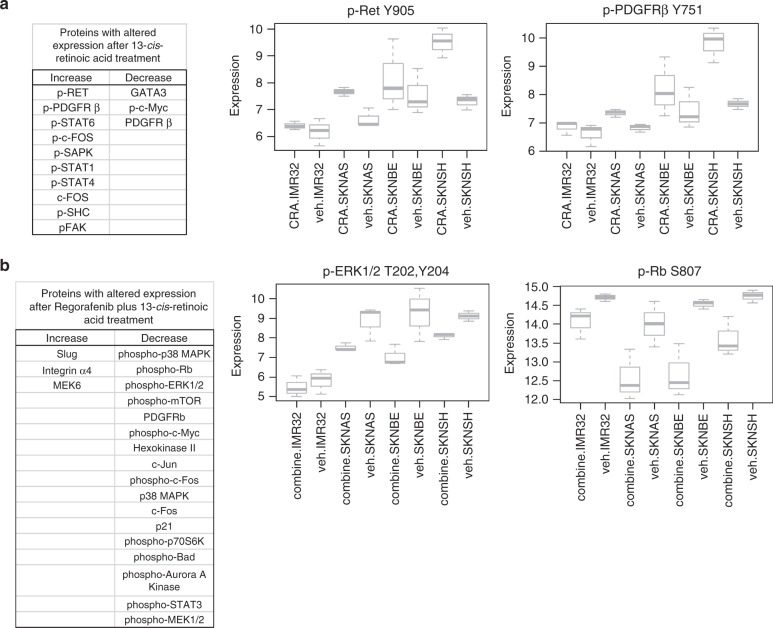
Regorafenib combined with 13-*cis*-retinoic acid alters protein expression and phosphorylation in neuroblastoma cells. Neuroblastoma cells were treated with vehicle alone, 5 μM 13-*cis*-retinoic acid alone or 5 µM regorafenib combined with 5 μM 13-*cis*-retinoic acid for 24 h. Lysates were collected and analysed by reverse-phase protein array (RPPA). **a** The results were analysed, and proteins whose levels or phosphorylation were significantly increased or decreased after treatment with 13-*cis*-retinoic acid when compared with vehicle alone are shown (left, *p* < 0.05 for each using ANOVA). Relative protein levels of phospho-RET and phospho-PDGFRβ were calculated as described, and are shown from cells treated with 13-*cis*-retinoic acid (CRA) and with vehicle alone (veh), with the results displayed separately for independent cell lines (right). **b** Proteins whose levels or phosphorylation were significantly increased or decreased after treatment with 5 µM regorafenib combined with 5 μM 13-*cis*-retinoic acid when compared with vehicle alone are shown (left, *p* < 0.05 for each using ANOVA). Relative protein levels of phospho-ERK1/2 and phospho-Rb were calculated as described, and are shown from cells treated with regorafenib combined with 13-*cis*-retinoic acid (combine) and with vehicle alone (veh), with the results displayed separately for independent cell lines (right).

To determine whether 13-*cis*-retinoic acid induced changes in the expression or activity of novel signalling pathways, we further analysed neuroblastoma cells treated with 13-*cis*-retinoic acid by RPPA as above. Seventeen proteins were found to have significant changes in expression or phosphorylation (*p* < 0.05) after 13-*cis*-retinoic acid treatment. Phosphorylation of RET, PDGFRβ, STAT6 and c-Fos, among others, was increased upon exposure to 13-*cis*-retinoic acid, while expression levels of GATA3 and phosphorylation of c-Myc were both reduced (Fig. 6a). Increased phosphorylation of RET has been demonstrated in response to 13-*cis*-retinoic acid in prior studies,^36^ establishing the validity of the RPPA results. Within these 17 proteins, four were found to have significant interaction between *MYCN* amplification and response to 13-*cis*-retinoic acid (ALK, p-Src, p–c-Fos and p-RET), with increased ALK expression but reduced RET and Src phosphorylation in *MYCN*-amplified cells compared with non-amplified cells. Three proteins (c-Fos, p-PDGFRβ and p-MAPK8) were found to have a significant interaction between known retinoic acid sensitivity and response to 13-*cis*-retinoic acid, with increased c-Fos expression and increased phosphorylation of PDGFRβ and MAPK8 (SAPK1/JNK1) in retinoic acid-sensitive cells compared with -resistant cells.

To determine whether the combination of regorafenib with 13-*cis*-retinoic acid induced additional changes in protein expression or activity, we further analysed neuroblastoma cells treated with regorafenib in combination with 13-*cis*-retinoic acid by RPPA as above. RPPA analysis of untreated neuroblastoma cells and of neuroblastoma cells treated with 5 μM regorafenib combined with 5 μM 13-*cis*-retinoic acid for 24 h identified 36 proteins with significant changes in expression (*p* < 0.05). Phosphorylation of p38 MAPK, Rb, ERK1/2, mTOR, c-Myc and c-Fos, among many others, was significantly reduced after treatment with regorafenib in combination with 13-*cis*-retinoic acid (Fig. 6b). Within these 36 proteins, 10 were found to have significant interaction between *MYCN* amplification and response to regorafenib combined with 13-*cis*-retinoic acid, with a larger decrease in c-Jun expression and c-Fos and Bad phosphorylation in response to the combination in *MYCN* non-amplified cells, and with reduced integrin α4 subunit expression and increased reduction in p70S6K and mTOR phosphorylation in *MYCN*-amplified cells compared with non-amplified cells (Supplemental Fig. 6). Two proteins (PDGFRβ and c-Myc) were found to have a significant interaction between known retinoic acid sensitivity and response to the combination of regorafenib with 13-*cis*-retinoic acid, with reduced expression of PDGFRβ and phosphorylation of c-Myc in retinoic acid-sensitive cells.

## Discussion

New treatment strategies are needed for children with high-risk and recurrent neuroblastoma, and therapies directed against a combination of relevant kinases and signalling pathways are likely to be effective against neuroblastoma. We have shown that the novel multikinase inhibitor regorafenib is effective against both neuroblastoma cell lines and tumours via induction of both apoptosis and cell cycle arrest, and we have further demonstrated that regorafenib inhibits both cell surface receptor kinases and a number of key intracellular signalling pathways. Our results also demonstrate the efficacy of the combination of regorafenib and 13-*cis*-retinoic acid. Lastly, our results identify critical intracellular signalling pathways in neuroblastoma cells that are inhibited or activated by regorafenib, 13-*cis*-retinoic acid and the combination of regorafenib with 13-*cis*-retinoic acid, and identify additional targets that may represent mechanisms of treatment resistance.

Neuroblastoma cells and primary tumours express a wide range of growth factors and receptor kinases that represent potential therapeutic targets,^6–20^ and regorafenib is a novel kinase inhibitor shown to inhibit a number of kinases relevant for neuroblastoma pathogenesis, including VEGFR1–3, c-Kit, TIE-2, PDGFRβ, FGFR1, RET, RAF1, BRAF and p38 MAPK.^21^ Both neuroblastoma cell lines and patient tumours have been shown to express the RET receptor tyrosine kinase,^37,38^ and RET expression and activity both induce neuroblastoma tumorigenesis in transgenic mice and enhance metastasis,^39,40^ suggesting that RET inhibition likely contributes to the efficacy of regorafenib against neuroblastoma. Other receptor kinases, including the vascular endothelial growth factor receptor (VEGFR) family and the platelet-derived growth factor-β receptor (PDGFRβ), have also been linked to neuroblastoma pathogenesis,^7,11,15,41,42^ suggesting that kinase inhibitors such as regorafenib that are able to target multiple critical kinases are likely to be effective against neuroblastoma, and further studies are needed to clarify the relative roles of these kinases in the responses of neuroblastoma tumours to regorafenib.

Ligand binding to cognate receptor tyrosine kinases leads to receptor activation and increased downstream intracellular signalling activity of the PI3K/mTOR/Akt, JAK/STAT and RAS/MAPK pathways, among many others. RAS/MAPK pathway signalling has been implicated in a variety of adult and paediatric malignancies,^43^ but the relevance of RAS/MAPK pathway activity in the development and progression of neuroblastoma tumours is not well understood. Neuroblastoma tumours have a paucity of oncogenic mutations, and activating RAS-MAPK pathway mutations are found in less than 5% of neuroblastoma patient tumours prior to the onset of treatment.^44,45^ However, mutations leading to increased RAS-MAPK pathway signalling are more common in tumours from patients with relapsed neuroblastoma,^46^ suggesting that RAS-MAPK pathway inhibitors are likely to be most effective in the setting of relapsed disease, and our data demonstrating that regorafenib is capable of inhibiting RAS-MAPK pathway signalling suggest a potential therapeutic role in children with relapsed neuroblastoma.

Our results further demonstrate alterations in the activity of numerous other signalling pathways in neuroblastoma cells after treatment with regorafenib, including the PI3K/mTOR/Akt and Fos/Jun pathways. The role of the PI3K/mTOR/Akt pathway in neuroblastoma pathogenesis has been well established,^47^ and early-phase clinical trials evaluating the efficacy of inhibitors of PI3K/mTOR/Akt signalling have demonstrated efficacy in children with neuroblastoma.^48–52^ The role of signalling through the Fos/Jun pathway in neuroblastoma pathogenesis is less well understood, although recent studies have identified a role for Jun kinase (JNK) signalling in neuroblastoma differentiation,^53^ and computational models of neuroblastoma demonstrated an association between JNK signalling and patient survival.^54^ The efficacy of inhibition of these pathways in other adult and paediatric cancer models has not previously been reported, and the specific roles of these pathways in the responses of neuroblastoma cells to regorafenib are unknown. Our results show the effects of prolonged, continuous regorafenib exposure on intracellular signalling in neuroblastoma cells, which may be more relevant in patients with neuroblastoma that are exposed to steady-state drug levels, and which suggests that the observed changes in the activity of signalling pathways may represent mechanisms of drug resistance as well. In addition, regorafenib may also inhibit other intracellular targets that were not evaluated in our screening studies, and additional studies are ongoing to delineate the specific mechanisms of regorafenib efficacy against neuroblastoma and other solid tumours.

Our results show that all tested neuroblastoma cell lines demonstrated responses to regorafenib within a relatively narrow range of doses, suggesting a potential common mechanism of drug response, or possibly multiple overlapping mechanisms. Regorafenib has demonstrated efficacy against both adult and paediatric solid tumours,^21,25,26,55–58^ and our results demonstrating regorafenib efficacy against neuroblastoma are similar to those previously observed for regorafenib in cell lines from many adult and paediatric tumours. In adults with advanced solid tumours, regorafenib therapy was well tolerated, and regorafenib has been FDA approved for the treatment of metastatic colorectal cancer, advanced gastrointestinal stromal tumours (GIST) and progressive hepatocellular carcinoma after prior sorafenib therapy.^21–24^ The safety and efficacy of regorafenib in children are currently being evaluated in an ongoing phase I clinical trial (NCT02085148), but commonly reported toxicities in adult patients at the approved dose of 160 mg daily for 3 consecutive weeks included hand–foot syndrome, nausea, diarrhoea, weight loss, fatigue and hypertension, with rare cases of severe liver toxicity.^59,60^ Peak regorafenib levels in the serum of adult patients taking the approved regorafenib dose exceeded 9 μM,^61,62^ which was within the range of our in vitro IC_50_ values. Therefore, effective levels of regorafenib are likely achievable in children with neuroblastoma, and regorafenib therefore represents a good candidate for further therapeutic investigation.

Retinoids are vitamin A analogues that induce tumour cell differentiation,^63^ and 13-*cis*-retinoic acid treatment of neuroblastoma cells in vitro both induces neuroblastoma cell differentiation and reduces neuroblastoma cell proliferation.^64–66^ 13-*cis*-retinoic acid is also currently a component of standard maintenance therapy in the most common protocols utilised for the treatment of children with high-risk neuroblastoma.^35^ Morphologic neuroblastoma cell differentiation induced by retinoids in vitro occurs over the course of 7–10 days, but the pathways activated prior to the morphologic differentiation are poorly understood. We have shown that activation of multiple kinases and signalling molecules, including RET, PDGFRβ, STAT6 and c-Fos, among others, precedes the morphologic differentiation induced by 13-*cis*-retinoic acid, and these signalling pathways may contribute to or regulate the subsequent morphologic changes. Furthermore, inhibition of these initially activated pathways may contribute to the efficacy of the combination of regorafenib and 13-*cis*-retinoic acid.

Recent studies have demonstrated a potential role for RET in the process of neuroblastoma differentiation, as 13-*cis*-retinoic acid treatment leads to increased RET expression, and RET inhibition has been shown to interfere with neuroblastoma differentiation induced by retinoic acid treatment.^37,67–69^ Neuroblastoma cell differentiation induced by retinoic acid treatment has also been shown to create a dependence on neurotrophin and glial-derived neurotrophic factor signalling, suggesting that retinoic acid treatment may sensitise neuroblastoma cells to inhibition of these pathways.^70,71^ We have previously identified synergistic efficacy of the combination of RET inhibition with 13-*cis*-retinoic acid.^29,36^ However, our results show that the combination of regorafenib with 13-*cis*-retinoic acid is additive, but did not reach statistical significance for synergy, possibly due to the effects of regorafenib on other kinase targets, such as the PI3K/mTOR/Akt and Fos/Jun pathways.

Our results demonstrate that regorafenib is effective against both neuroblastoma cell lines and xenograft tumours, and suggest that regorafenib may be an effective treatment for children with neuroblastoma, and the inhibition of multiple intracellular signalling pathways likely underlies the efficacy of regorafenib against neuroblastoma. With the established roles of RET and RAS/MAPK signalling in neuroblastoma pathogenesis and with the efficacy of regorafenib established in these and other studies, additional preclinical and clinical testing of regorafenib in children with neuroblastoma is clearly warranted. Furthermore, the combination of regorafenib and 13-*cis*-retinoic acid is likely to be effective and could be employed either for maintenance therapy of newly diagnosed high-risk neuroblastoma or for treatment of relapsed disease.

## Supplementary information

Supplementary files

## Data Availability

All data generated or analysed during this study are included in this published article (and the Supplementary information files).
