# Supplementary material for: Regorafenib is effective against neuroblastoma in vitro and in vivo and inhibits the RAS/MAPK, PI3K/Akt/mTOR and Fos/Jun pathways
Source: Br J Cancer. 2020 May 27;123(4):568–79. doi: 10.1038/s41416-020-0905-8 (PMC7434894; doi:10.1038/s41416-020-0905-8)
Supplement: Supplementary file 1 — Supplementary files [file 41416_2020_905_MOESM1_ESM.doc]

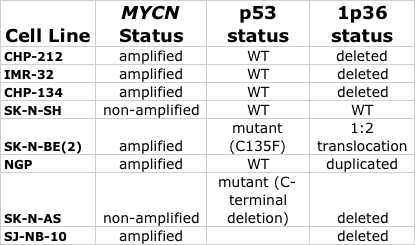


***Supplemental Table 1. Characteristics of Neuroblastoma Tumor Cell Lines.***

***
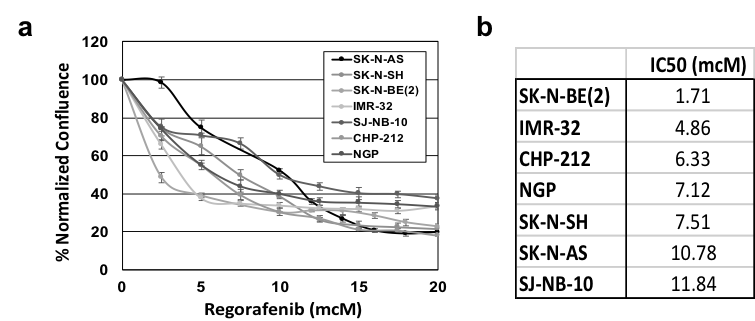
***

***Supplemental Figure 1. Regorafenib Reduces Neuroblastoma Cell Confluence.***

***
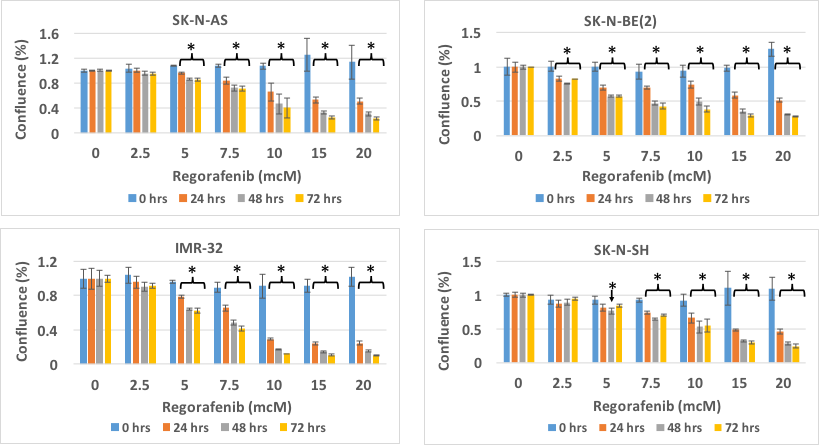
***

***Supplemental Figure 2. Regorafenib Reduces Neuroblastoma Cell Confluence.***


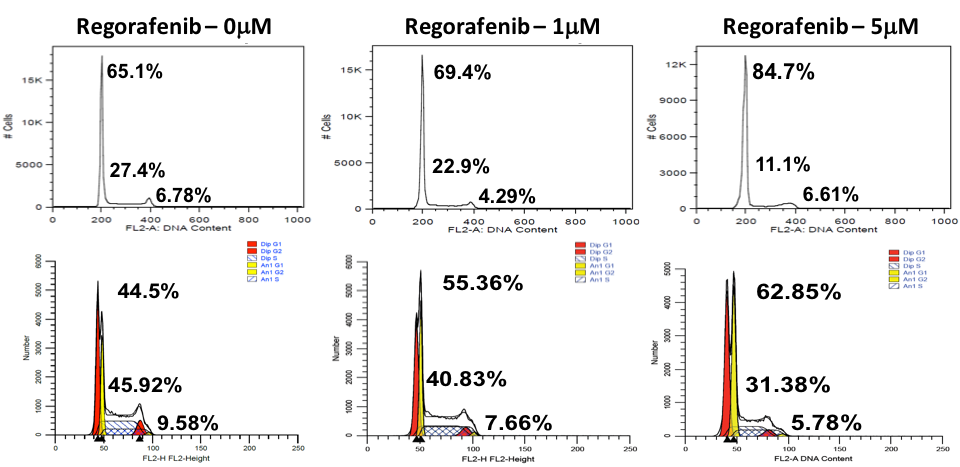


***Supplemental Figure 3. Regorafenib Induces Cell Cycle Arrest in Neuroblastoma Cells.***


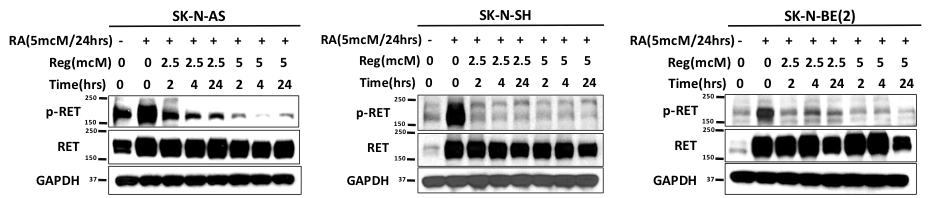


***Supplemental Figure 4. Regorafenib Inhibits 13-cis-Retinoic Acid-induced RET phosphorylation in Neuroblastoma Cells.***


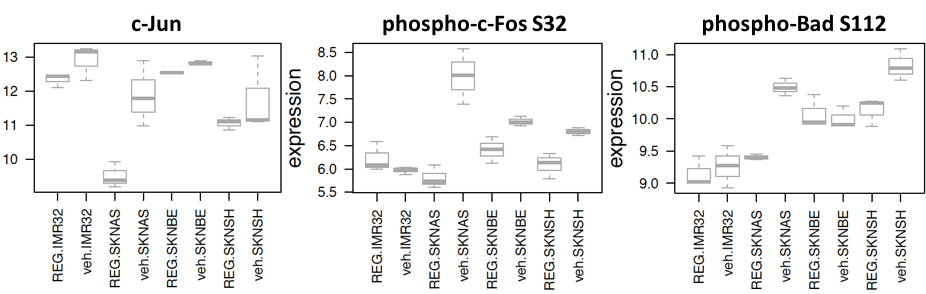


***Supplemental Figure 5. Regorafenib Treatment Alters Protein Expression and Phosphorylation in MYCN-amplified and MYCN-nonamplified Neuroblastoma Cells***.


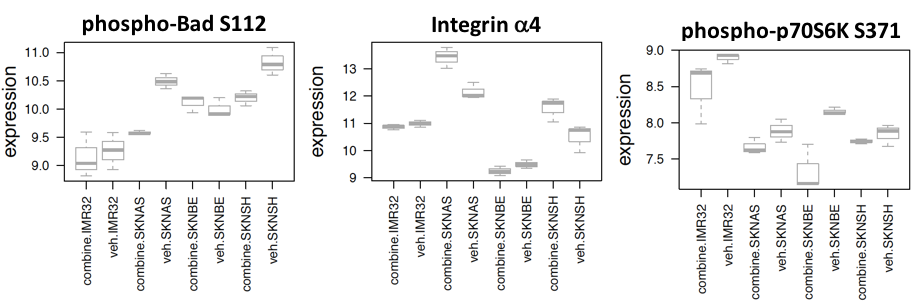


***Supplemental Figure 6. Regorafenib Combined with 13-cis-Retinoic Acid Treatment Alters Protein Expression and Phosphorylation in MYCN-amplified and MYCN-nonamplified Neuroblastoma Cells.***

***Supplementary Figure Legends***

***Supplemental Figure 1. Regorafenib Reduces Neuroblastoma Cell Confluence.*** (A) Neuroblastoma cell lines (SK-N-AS, SK-N-SH, SK-N-BE(2), IMR-32, NGP, CHP-212, and SJ-NB-10) were treated with regorafenib for 72 hours and were analyzed by continuous live cell imaging. The normalized percentage confluence was calculated for each line, and mean average values from triplicate experiments were plotted against regorafenib concentrations. (B) IC50 values were calculated for each tested neuroblastoma cell line.

***Supplemental Figure 2. Regorafenib Reduces Neuroblastoma Cell Confluence.*** Neuroblastoma cell lines were treated with regorafenib and were analyzed by continuous live cell imaging at 24, 48, and 72 hours. The normalized percentage confluence was calculated for each cell line at each time point, and mean average values from triplicate experiments were plotted against regorafenib concentrations. Final results were compared using Student's t-tests (* p<0.005 for IMR-32; *p<0.01 for SK-N-AS, *p<0.05 for SK-N-BE(2) and SK-N-SH)

***Supplemental Figure 3. Regorafenib Induces Cell Cycle Arrest in Neuroblastoma Cells.***  SK-N-SH (top) and IMR-32 (bottom) neuroblastoma cells were treated with 1μM or 5μM regorafenib and analyzed after 24 hours by flow cytometry for cell cycle progression. Percentages of cells in G1, S, and G2/M phases are shown for each experiment.

***Supplemental Figure 4. Regorafenib Inhibits 13-cis-Retinoic Acid-induced RET phosphorylation in Neuroblastoma Cells.*** Neuroblastoma cells were treated with vehicle alone or 5μM 13-*cis*-retinoic acid (RA) with or without added regorafenib for 2, 4, and 24 hours and the protein lysates were analyzed by Western blot for total and phosphorylated RET (RET, p-RET). GAPDH was used as a loading control.

***Supplemental Figure 5. Regorafenib Treatment Alters Protein Expression and Phosphorylation in MYCN-amplified and MYCN-nonamplified Neuroblastoma Cells.***  *MYCN*-amplified (IMR-32, SK-N-BE(2)) and MYCN-nonamplified (SK-N-AS, SK-N-SH) neuroblastoma cells were treated with either vehicle or 5μM regorafenib for 24 hours. Lysates were collected and analyzed by Reverse Phase Protein Array (RPPA). Relative protein levels of identified proteins were compared between *MYCN*-amplified and non-amplified cell lines to determine whether *MYCN* amplification had an effect on observed changes in protein levels. Relative protein levels for c-Jun, phospho-c-Fos (S32), and phospho-Bad (S112) were calculated as described and are shown in regorafenib-treated (REG) and vehicle-treated (veh) neuroblastoma cells, with results displayed separately for independent cell lines (p<0.05 by ANOVA for levels in *MYCN*-amplified cells compared to *MYCN*-nonamplified cells).

***Supplemental Figure 6. Regorafenib Combined with 13-cis-Retinoic Acid Treatment Alters Protein Expression and Phosphorylation in MYCN-amplified and MYCN-nonamplified Neuroblastoma Cells.***  *MYCN*-amplified (IMR-32, SK-N-BE(2)) and *MYCN*-nonamplified (SK-N-AS, SK-N-SH) neuroblastoma cells were treated with either vehicle or 5μM regorafenib combined with 5μM 13-*cis*-retinoic acid (CRA) for 24 hours. Lysates were collected and analyzed by Reverse Phase Protein Array (RPPA). Relative protein levels of identified proteins were compared between *MYCN*-amplified and non-amplified cell lines to determine whether *MYCN* amplification had an effect on observed changes in protein levels. Relative protein levels for phospho-Bad, integrin α4, and phospho-p70S6K were calculated as described and are shown in cells treated with the combination (combine) and vehicle-treated (veh) neuroblastoma cells, with results displayed separately for independent cell lines (p<0.05 by ANOVA for levels in *MYCN*-amplified cells compared to *MYCN*-nonamplified cells).
